# Contextual Cueing Effect in Spatial Layout Defined by Binocular Disparity

**DOI:** 10.3389/fpsyg.2017.01472

**Published:** 2017-08-31

**Authors:** Guang Zhao, Qian Zhuang, Jie Ma, Shen Tu, Qiang Liu, Hong-jin Sun

**Affiliations:** ^1^Research Center of Brain and Cognitive Neuroscience, Liaoning Normal University Dalian, China; ^2^Department of Psychology, Institute of Education, China West Normal University Nanchong, China; ^3^Department of Psychology, Neuroscience and Behaviour, McMaster University, Hamilton ON, Canada

**Keywords:** contextual cueing effect, depth vision, binocular disparity, spatial layout, attentional guidance

## Abstract

Repeated visual context induces higher search efficiency, revealing a contextual cueing effect, which depends on the association between the target and its visual context. In this study, participants performed a visual search task where search items were presented with depth information defined by binocular disparity. When the 3-dimensional (3D) configurations were repeated over blocks, the contextual cueing effect was obtained (Experiment 1). When depth information was in chaos over repeated configurations, visual search was not facilitated and the contextual cueing effect largely crippled (Experiment 2). However, when we made the search items within a tiny random displacement in the 2-dimentional (2D) plane but maintained the depth information constant, the contextual cueing was preserved (Experiment 3). We concluded that the contextual cueing effect was robust in the context provided by 3D space with stereoscopic information, and more importantly, the visual system prioritized stereoscopic information in learning of spatial information when depth information was available.

## Introduction

In a natural environment, objects hardly exist in isolation. When we interact with objects in a familiar environment, other objects in the environment might serve as a context that allows us to process visual stimuli more efficiently. Previous research has shown that, in visual search, repeated configurations in which search items (the target and distracters) in a given scene appeared in constant locations over blocks could induce faster responses than random configurations in which the target was presented with a variant configuration. This contextual benefit of invariant configurations has been referred to as the contextual cueing effect (Chun and Jiang, 1998; Chun and Phelps, 1999; Chun, 2000; Olson et al., 2001; Kunar et al., 2006; Zhao et al., 2012). Interestingly, while the contextual cueing effect was obtained, participants were not aware of the repetition of the configurations, which indicated that an implicit learning mechanism was involved in the contextual cueing paradigm (Chun and Jiang, 1998) (but see also Smyth and Shanks, 2008). It is generally considered that the contextual cueing effect was generated on the acquisition of covariations between the location of the target and location of the distractor items. The investigation of contextual cueing effect can help us better understand the learning of critical contextual information and how context guides the deployment of attention (Chun, 2000; Olson and Chun, 2002).

One distinct feature of the contextual cueing effect is said to be its ecological validity in real-world settings, since objects in the world are often present in a given context (Chun and Jiang, 1998; Chun, 2000; Chua and Chun, 2003; Torralba et al., 2006). In fact, invariant contextual information is ubiquitous in real world 3D scenes. The classical paradigm reported by Chun and Jiang (1998) focused on the invariance in spatial relations between target and context in 2-dimensional space. Moreover, the stimuli used in most of the subsequent studies featuring contextual cueing paradigms involved only 2D displays and lacked depth information. However, the learning of spatial relations between target and distractor arrays in a 3-dimensional space has not been well studied.

Studies of visual perception have demonstrated the importance of depth information in the perception of objects and scenes. In fact, researchers have demonstrated that depth cues can over-ride some salient 2-D cues in influencing object recognition (Howard and Rogers, 1995; Liu and Ward, 2006; Akhavein and Farivar, 2017). However, the role of depth information remains unclear in the context of implicit learning. In the contextual cueing paradigm, it remains an open question whether the learning of the search context can be extended to the depth dimension.

Chua and Chun (2003) presented computer-rendered artificial scenes that used pictorial cues to give an impression of apparent depth. An array of objects was positioned on a ground plane and apparent depth information was provided through linear perspective cue. Using such display, they successfully obtained the contextual cueing effect. Note that in this case, objects further in depth along the *z* axis would appear higher along the *y* axis of the fronto-parallel plane (display screen). Therefore, the effect found here could in principle still be considered as an effect in 2D space.

Binocular disparity is another important cue to depth (He and Nakayama, 1992; Qian, 1997; Finlayson et al., 2012). It is well known that the human brain can process information about depth using binocular disparities alone (Barlow et al., 1967; Johnston et al., 1994). If depth information defined by binocular disparity is introduced into the contextual cueing paradigm for repeated configurations, the invariant relation between target and context could be defined by the combined location information in the fronto-parallel plane and in-depth (*x, y*, and *z* coordinate).

Kawahara (2003) attempted to investigate the contextual cueing effect with a stereoscopic display. The experiments presented stimulus items in two depth planes defined by binocular disparity and instructed participants to attend to items in one depth plane and ignore items in the other plane. This design was similar in principle to Jiang and Chun (2001), in which visual context was defined by items of a particular color while items of another color were ignored. In fact, both studies explored selective attention on the role of global and local contexts for the contextual cueing effect. In addition, in the study by Kawahara (2003), participants were required to group items into two parts by binocular disparity, but did not need to search across different depth planes. Real world scenes are rich in depth information and spatially continuous rather than isolated in one depth plane (Wolfe, 1994). The task in Kawahara (2003), although defined by binocular disparity, was limited in spatial continuity in depth domain, thus could still be considered as a 2D task.

Tsuchiai et al. (2012) used a search task presented in stereoscopic depth to examine the contextual cueing effect in 3D space. There was no detailed description of the exact 3D manipulation in that study, but it is likely that the stimulus items were randomly scattered in different depth planes. They demonstrated contextual cueing effect using this stereoscopic display, however, no attempt was made in examining whether stereoscopic information actually contributed to the contextual cueing effect.

It is important to point out that, for typical repeated scenes in a stereoscopic display, the invariant relation between target and context, in theory, can still be defined through 2D information (displacement on x and/or y coordinate) alone. The 3d information (displacement on *z* axis) of the items, if repeated, would be redundant in informing the structure of the layout.

In the present study, we set out to demonstrate again that the contextual cueing effect could be obtained in an invariant target-distractors association defined by both 2D and 3D location. More importantly, we went beyond what Tsuchiai et al. (2012) showed by examining whether invariant relation in depth between target and distractors was necessary for the contextual cueing effect when the disparity information of the distractors was available.

In Experiment 1, we varied the binocular disparity of the distracter items in both predictive and random displays and held the disparity information as well as 2D information constant for the predictive displays. The results demonstrated that the contextual cueing effect could be obtained in visual search in depth.

Although the results showed the contextual cueing effect in Experiment 1, the participants may have solely relied on 2D information. In Experiment 2, we examined whether participants actually used the disparity information. Specifically, we tested whether the contextual cueing effect would disappear when the depth information of distracter items was randomized across blocks in predictive configurations while the 2D information of the layout was held constant. The results showed a lack of contextual cueing effect suggesting that participants might use the disparity information to learn the predictive display in Experiment 1.

However, the variation of disparity information in Experiment 2 led to a small displacement in the horizontal axes in the 2D plane as well. Thus in Experiment 3’s predictive displays condition, we introduced a comparable 2D displacement in the distracter items while maintaining the disparity information constant across blocks. The results showed that the contextual cueing effect resumed, suggesting that the lack of contextual cueing effect in Experiment 2 was not due to the small 2D displacements created in the process of variating disparity in Experiment 2. Consequently, the data suggests that participants indeed relied on disparity information when it was available to achieve the contextual cueing effect.

For the contextual cueing effect in a 2D display, it has been demonstrated that attentional guidance is the mechanism in which the repeated context guides participants’ attention toward the target (Chun and Jiang, 1998). The evidence of such a mechanism came from the slope and intercept of the RT × Set Size function when the set size of the search items were varied in the experiment. It was found that the slope was lower in repeated displays compared to random displays, but such pattern was not seen for intercept. However, results from other studies showed the such effect of improved search efficiency has been less consistent (Kunar et al., 2007), suggesting that attentional guidance might not be the only mechanism for the contextual cueing effect.

The present study also investigated the mechanism of the contextual cueing effect generated in a 3D setting. We varied the set size of configuration in all experiments. We fitted a line to the RT × Set Size function, and analyzed the slope and intercept of the fitting line. Based on the predictions of previous studies, if contextual cueing was driven by attentional guidance, there would be a downward trend in the slope and slope for repeated scene will be lower; otherwise, if contextual cueing was sourced by non-search factors (the perceptual recognition processing or the response selection processing), such pattern of results would not been seen (Chun and Jiang, 1998; Jiang et al., 2005; Zhao et al., 2012).

## Experiment 1

### Methods

#### Participants

Twenty undergraduate students (9 males and 11 females, mean age = 21 years) participated in the experiment and received a small payment. All participants were right-handed, with normal or corrected-to-normal vision. They first performed a task to ensure they could perceive 3D structure with stereo goggles. Participants were naïve to the experimental hypotheses before they accomplished the experiment. All participants provided informed written consent prior to the experiment. The experimental protocol was approved by the Ethics Committee of Liaoning Normal University, China. The methods were performed in accordance with the approved guidelines. One participant was excluded from analyses because of incomplete data collection. All participants gave written informed consent in accordance with the Declaration of Helsinki.

#### Apparatus and Stimuli

The experiment was conducted in a quiet and dark room (15 m × 7 m). Stimuli were projected onto a film screen using a rear-projection device (JVC projector DLA-SX21). The screen size was 246 cm × 182 cm and the image resolution was 1024 pixels × 768 pixels with a frame rate of 60 Hz. Participants were asked to maintain their body steadily and viewed the screen from a distance of 3 m. Participants wore stereo goggles that provided two 2D images with a horizontal offset to elicit the perception of 3D. To make participants at ease and to provide a better viewing posture, stimuli were presented in the lower part of the central axis. That is, the distance between the center position of stimuli and the floor was 150 cm.

**FIGURE 1:**
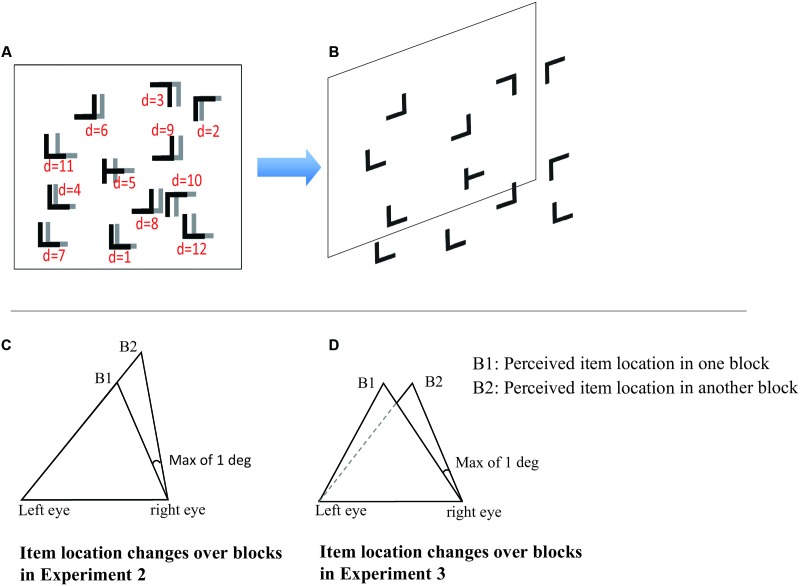
A schematic illustration of the search display. **(A)** Stimuli presented in two eyes, respectively. The stimulus presented to one eye is illustrated in black letters and that presented to the other eye is illustrated in gray letters. The d stands for the distance of binocular disparity for each stimulus item (the distances are examples of different depth ranges). For predictive configurations, the distances of elements remain constant across blocks in Experiments 1 and 3, but not in Experiment 2; for random configurations, all the distances of distracters are randomized. **(B)** Illustration of stereoscopic depth for the search display. **(C)** An illustration of disparity variation across blocks for predictive configurations in Experiment 2. B1 stands for perceived depth location of the stimulus in one block, B2 stands for the same item perceived depth location in another block. Other eye (e.g., right eye) were randomized within [0.1°, 1°]. It is perceived with variant depth between B1 and B2. **(D)** An illustration of invariant disparity across blocks for predictive configurations in Experiment 3. The item is presented in both eyes are translated synchronously. It is perceived with difference fronto-plane but invariant depth in B1 and B2.

Within each display, the target was a letter “T” rotated by 90° either clockwise or counter-clockwise, and the distracters were letter “L”s rotated randomly by 0, 90, 180, or 270°. The two lines in each stimulus item were of equal length and with the length of 2.3° and the line thickness of 0.1°. Two set sizes were used (7 distracters and 1 target for set size 8 and 11 distracters and 1 target for set size 12). All the search items were randomly distributed over an invisible array of 8 × 6 locations (x and y coordinates). The array grid subtended 34.4° × 25.8° of visual angle. To avoid the formation of collinearities among the stimulus items, the position (x and y coordinates) of each item had a slight random displacement within a range of [0°, 0.8°] in the vertical and horizontal axes. The background of the screen was gray, and the stimuli were always black. To produce stereovision, binocular disparity (in z coordinate) in the search items was presented over two eyes. The disparity of each search item was randomized within a range of [0.1°, 1°], making a perceived depth distance away from participants within a range of [3.27 m, 14.18 m]. An example stimulus is illustrated in **Figures 1A,B**.

#### Design

Three within-subject factors were included: configuration (predictive vs. random), epoch (1∼7 epochs) and set size (8 vs. 12). There were two types of configurations, predictive and random. Each predictive configuration was presented once in a block and reoccurred in every block throughout the whole experiment. In particular, for the predictive configurations, both the 2D locations (*x*- and *y*-values) and the disparity values (*z*-value) of all the items were repeated across blocks. For the random configurations, both the 2D locations and the disparity of each item were randomized except that the same set of target location (*x-, y*-, and *z*-values) was used in all blocks. We also balanced, within and between configurations, possible target locations across four quadrants and in different eccentricities. The predictive and random displays have different sets (but in equal number) of possible target locations.

The entire experiment consisted of 28 blocks of 16 trials (8 random and 8 predictive trials, each contained 4 scenes with the set size of 8, and 4 scenes with the set size of 12) with a total of 448 experimental trials. Two different set sizes (8 and 12) were randomized within a block. To enhance the statistical power, in data analysis, 4 blocks in a row were grouped and averaged into one epoch, which resulted into 7 epochs as the time window.

#### Procedure

Each trial started with a centrally presented fixation cross “+” (500 ms), followed by the search display. Participants were asked to search for the target (left or right orientated “T”) and responded upon detection as quickly and accurately as possible. Participants were asked to respond by pressing one of two keys: the “F” key for the left rotated target and the “J” key for the right rotated target. The trial would terminate when no response was detected for 10 s. After the participant had responded, a blank gray display was shown for 200 ms and then a screen with the word “Next” appeared for 200 ms indicating the onset of the next trial. Before the formal test, participants performed one practice block of 20 trials to get familiar with the task.

### Results

The overall mean accuracies were 99.05% in each conditions, and showed no significant effects (all *p*’s > 0.110). This pattern of results for mean accuracy was similar for Experiment 2 and 3, we thus will not describe accuracy results for subsequent experiments and will mainly focus on the mean RT data in the data analysis.

**FIGURE 2:**
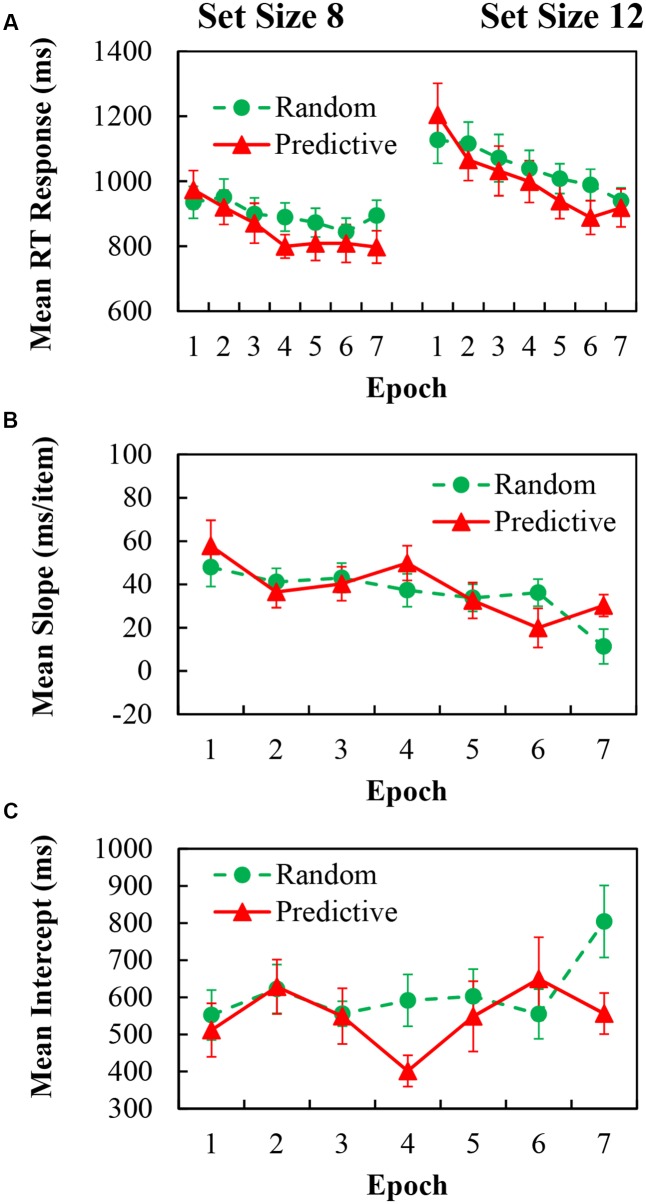
**(A)** Mean correct RTs in each epoch for predictive (red) and random (green) configurations in set size 8 (Left) and set size 12 (Right) of Experiment 1. **(B)** Search slopes (ms/item) for predictive (red) and random (green) configurations over epoch of Experiment 1. **(C)** Intercepts (ms) for predictive (red) and random (green) configurations over epoch of Experiment 1.

For the mean RT data, trials with incorrect responses and RTs below 200 ms or above 4000 ms (representing less than 0.6% of all outliers and errors) were excluded. The mean RTs for predictive and random configurations with epochs for set sizes of 8 and 12 are shown in **Figure 2** (left and right panels, respectively). The mean RTs were analyzed in a repeated measures ANOVA of 2 (configuration) × 7 (epoch) × 2 (set size). There were significant main effects of configuration [*F*(1,18) = 8.227, *p* = 0.01, η^2^ = 0.314], indicating 39.19 ms faster RTs in predictive configurations than in random configurations; and epoch [*F*(6,108) = 17.194, *p* < 0.001, η^2^ = 0.489], indicating 172.84 ms reduction of RTs over time; and set size [*F*(1,18) = 74.735, *p* < 0.001, η^2^ = 0.806], indicating 147.92 ms faster search times for the larger set size. Further, the two-way interaction was significant for configuration × epoch [*F*(6,108) = 4.976, *p* < 0.001, η^2^ = 0.217]; *Post hoc* simple effects analysis demonstrated that predictive configurations needed longer search times in the first two epochs (*p*’s < 0.041), but the situation was reversed from 4th epoch to the last epoch (*p*’s < 0.05), indicating greater contextual benefit of predictive configurations as the epoch progressed. The two-way interaction of set size × epoch [*F*(6,108) = 4.251, *p* = 0.015, η^2^ = 0.191] was also significant. *Post hoc* simple effects analysis demonstrated that RTs in set size 8 were all significantly faster than in set size 12 in each epoch session (*p*’s < 0.001), indicating that more search times were needed for the larger set size as the epoch session progressed. However, the two-way interaction of set size × configuration, and the three-way interaction of configuration × epoch × set size, were not reliable.

To obtain the entire learning effect, we analyzed the cueing effect, in which we collapsed set size condition of reaction times and then compute the difference between predictive and random configurations. A one-way ANOVA for the Cueing effect of the learning epochs showed that there was significant main effect of epoch [*F*(6,108) = 4.976, *p* < 0.001, η^2^ = 0.217], indicating learning effect was obtained.

We further examine how context influences the efficiency of search using target slope measures as function of set size. Search slopes and intercepts were derived from each individual’s mean data. The slope as a function of configuration and epoch are shown in **Figure 2B**, the corresponding intercept are in **Figure 2C**. The slope data were analyzed in a repeated measures ANOVA of 2 (configuration) × 7 (epoch). There was a significant main effect of Epoch [*F*(6,108) = 4.251, *p* < 0.001, η^2^ = 0.191]. However, the main effect of configuration and the two-way interaction were not reliable. Analyzing the intercept data, the main effects of configuration and epoch were not reliable, but the two-way interaction of configuration × epoch was marginally significant [*F*(6,108) = 1.881, *p* = 0.091, η^2^ = 0.095]. Note that the slope was greater in predictive configurations than in random configurations, but the intercept was just the opposite.

### Discussion

Experiment 1 examined whether contextual cueing effect could take place in scenes containing stimulus items presented in different depth planes through binocular disparity. The results showed that the response RTs were significantly faster in predictive configuration than in random configuration as the learning progressed, indicating that contexts defined by depth can induce contextual cueing. The results replicated the general pattern of results by Tsuchiai et al. (2012), suggesting that learned associations between visual context and target presented in 3D space can facilitate search performance.

The stimuli in both predictive and random configurations were scattered similarly on different depth planes by manipulating the binocular disparity of elements projected in two eyes. Moreover, the possible target locations were also matched across the configurations. Thus any difference in results should be attributed to the repetition of the contextual information only.

## Experiment 2

Even though the results of Experiment 1 suggested that participants could learn the contextual items presented in different depths, this result cannot exclude the possibility that participants only use the spatial displacement between items in the fronto-parallel plane to learn the spatial layout. To investigate whether disparity information was indeed learned as part of the context in Experiment 1, in Experiment 2, the items in the predictive displays no longer maintained the same disparity information across blocks even though the 2D displacement between items remained largely invariant.

### Methods

#### Participants

Information about the participants were identical to that of Experiment 1 except that 21 undergraduate students (10 males and 11 females, mean age = 21 years) participated in the experiment.

#### Apparatus, Stimuli, and Procedure

Apparatus, stimuli, and procedure were the same as in Experiment 1, except that in each predictive configuration, the disparity of each item presented between left eye and right eye was not constant over blocks, instead, disparity was randomly varied within a range of [0.1°, 1°]. The target location (*x*- and *y*-values) remained largely invariant. Specifically, we kept 2D locations (x, y coordinates) of distracters presented in one eye (e.g., left eye) constant and the 2D locations of distracters presented in the other eye (e.g., right eye) with a relatively small horizontal displacement over blocks created due to the disparity variation (**Figure 1C**). Note that in this experiment, we continue to use the term “predictive display” to follow the conventional use of this term referring to a layout that is repeated in 2d space. In fact, the 3D layout in this experiment was not repeated.

### Results

**FIGURE 3:**
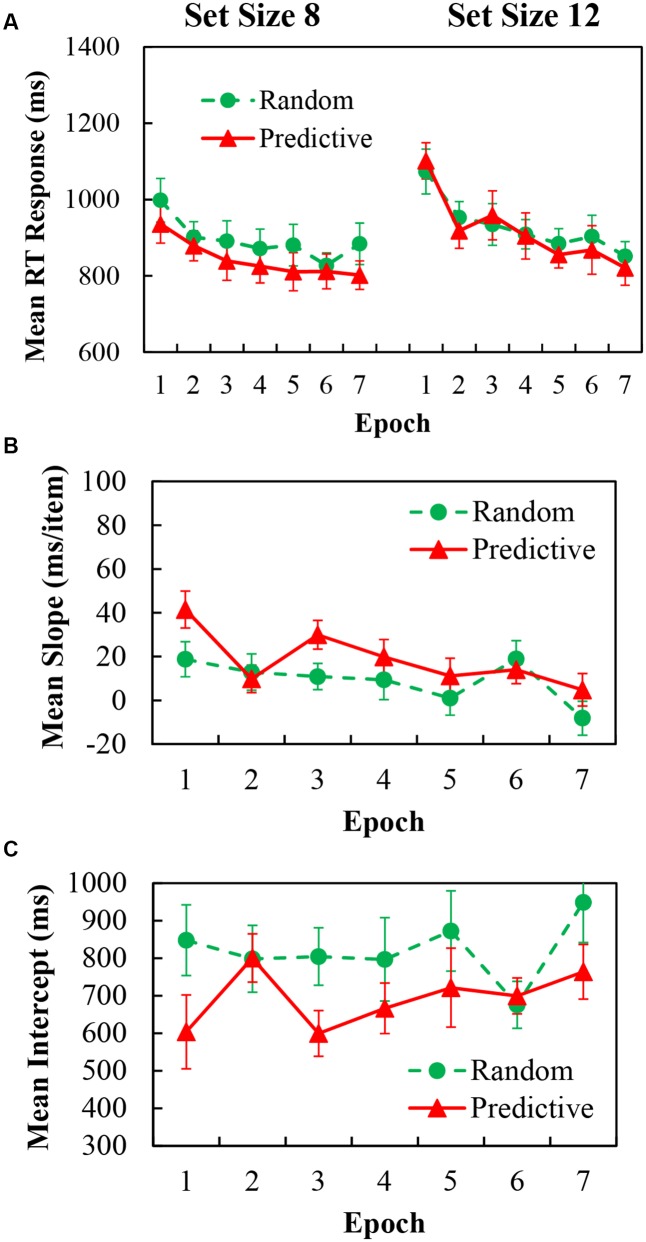
**(A)** Mean correct RTs in each epoch for predictive (red) and random (green) configurations in set size 8 (Left) and set size 12 (Right) of Experiment 2. **(B)** Search slopes (ms/item) for predictive (red) and random (green) configurations over epoch of Experiment 2. **(C)** Intercepts (ms) for predictive (red) and random (green) configurations over epoch of Experiment 2.

For the analysis of mean RT data, again trials with incorrect response and RTs below 200 ms or above 4000 ms (less than 0.3% of the data) were excluded. The mean RTs for predictive and random configurations with epochs for set sizes of 8 and 12 are shown in **Figure 3**. A 2 (configuration) × 7 (epoch) × 2 (set size) repeated measures ANOVA demonstrated significant main effects of configuration [*F*(1,20) = 16.734, *p* < 0.001, η^2^ = 0.456], indicating 30.92 ms faster RTs in predictive configurations than in random configurations; epoch [*F*(6,120) = 12.580, *p* < 0.001, η^2^ = 0.386], indication 187.54 ms RTs increased over time; and set size [*F*(1,20) = 18.106, *p* < 0.001, η^2^ = 0.475], indicating 55.64 ms greater RTs for the larger set size. The two-way interaction was significant for set size × epoch [*F*(1,20) = 3.170, *p* = 0.021, η^2^ = 0.137]; *Post hoc* simple effects analysis demonstrated that the larger set size needed longer RTs in the first epoch [*F*(1,20) = 18.54, *p* < 0.001], the 3rd epoch [*F*(1,20) = 25.27, *p* < 0.001], the 4th epoch [*F*(1,20) = 6.11, *p* = 0.023] and the 6th epoch [*F*(1,20) = 6.24, *p* = 0.021]. The two-way interaction was significant for set size × configuration [*F*(6,120) = 5.233, *p* = 0.033, η^2^ = 0.207]; *Post hoc* simple effects analysis demonstrated that in set size 8, predictive configurations have less RTs than random configurations [*F*(1,1) = 36.88, *p* < 0.001]; but this difference was limited in set size 12. More importantly, two-way interaction for configuration × epoch [*F*(6,120) = 0.827, *p* = 0.478, η^2^ = 0.040] were not significant, indicating that little contextual cueing effect was obtained as the epoch session progressed. Moreover, the three-way interaction between configuration × epoch × set size were not significant either. One way ANOVA for the Cueing effect of the learning epochs showed no main effect of epoch [*F*(6,120) = 0.827, *p* = 0.478, η^2^ = 0.040], indicating no learning effect was obtained.

The slope as a function of configuration and epoch are shown in **Figure 3B**, the corresponding intercept are in **Figure 3C**. For the slope data, there was a significant main effect of configuration [*F*(1,20) = 5.223, *p* = 0.033, η^2^ = 0.207], and epoch [*F*(6,120) = 3.170, *p* = 0.006, η^2^ = 0.137]. The two-way interaction was not reliable. For the intercept data, there was a significant main effect of configuration [*F*(1,20) = 10.560, *p* = 0.004, η^2^ = 0.346], the main effect of epoch and the two-way interaction of configuration × epoch were not reliable.

### Discussion

In Experiment 2, the experimental stimuli were almost identical to those used Experiment 1, the only difference being the randomized disparities of the distractor items in predictive configurations per trial, the 2D views were exactly invariant on the left eye and largely invariant on the right eye. It was hypothesized that if took advantage of the invariant 2D views of the distractor layout in the predictive displays and paid no attention to the depth information, the contextual cueing effect would not be affected during target location. However, the results of Experiment 2 showed no significant interaction between configuration and epoch. Although the main effect of configuration and epoch were significant in set size 8, the configuration × epoch interaction was not. This was mainly due to a 60 ms difference consistently observed across all epochs. This suggests that participants did not learn the predictive configuration due to variability of the stereoscopic information in the configuration. For set size 12, the main effect of configuration and epoch and the configuration × epoch interaction were not significant either, suggesting that the predictive configuration was not learned.

## Experiment 3

The results of Experiment 2 suggested that randomized depth cue in predictive displays created interference in contextual cueing in this experiment. Thus, depth cues defined by binocular disparity could contribute to the contextual cueing effect found in Experiment 1. However, someone could object that in the process of randomizing disparity, a tiny displacement (within a range of [0.1°, 1°]) in the horizontal axes of the distracter item for the right eye was created. One might argue that this displacement in the right eye might prevent the participants learning the invariant structure of the distracter layout for the predictive contexts. In Experiment 3, we explore whether such small 2D displacement could lead to a cost in contextual cueing effect. if the contextual cueing effect was obtained in Experiment 3, we could exclude the possibility that the interference of contextual cueing effect found in Experiment 2 was resulted from the tiny displacement of stimuli items.

### Methods

#### Participants

Information about the participants were identical to that of Experiment 1 except that 21 undergraduate students (10 males and 11 females, mean age = 21 years) participated in the experiment.

#### Apparatus, Stimuli, and Procedure

Apparatus, stimuli, and procedure were the same as in Experiment 1, except that, in predictive configurations, while the disparity for each item was kept constant, the image of each item presented in left eye and right eye was synchronously displaced in the fronto-parallel plane (x and y coordinates) within a range of [0.1°, 1°] across blocks. In other words, in predictive configurations, all the stimuli scattered on different depth planes and maintained the same depth information (z coordinate) when it recurred in the next block, but in the fronto-parallel plane the 2D locations (x and y coordinates) had a tiny displacement in the horizontal axes (**Figure 1D**).

### Results

**FIGURE 4:**
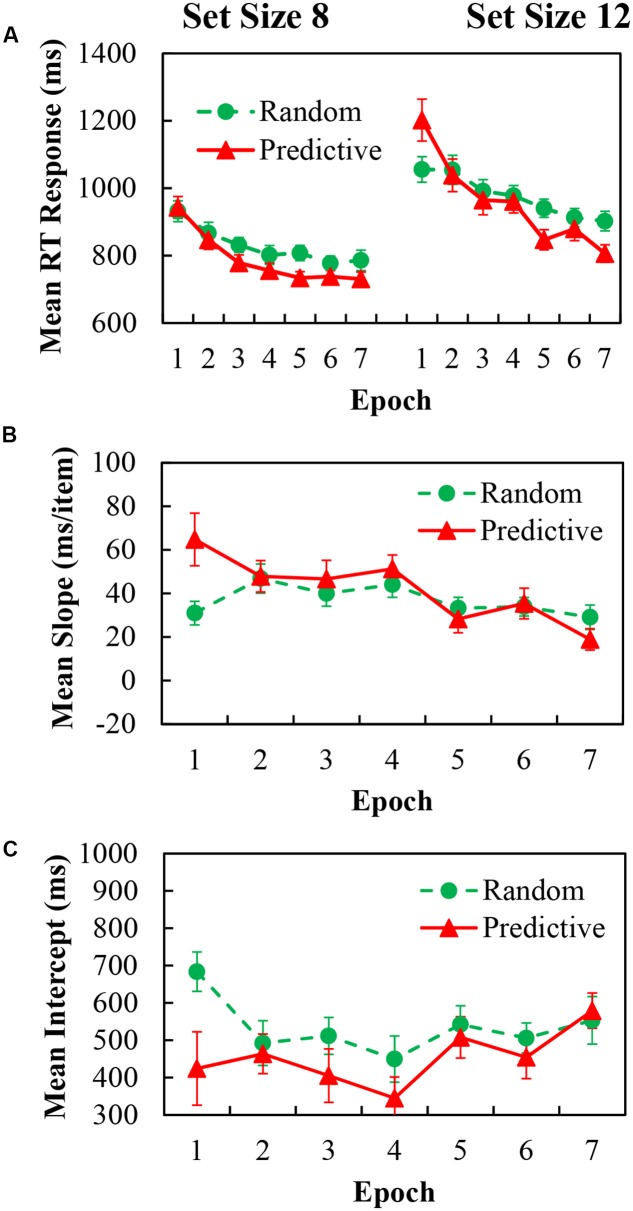
**(A)** Mean correct RTs in each epoch for predictive (red) and random (green) configurations in set size 8 (Left) and set size 12 (Right) of Experiment 3. **(B)** Search slopes (ms/item) for predictive (red) and random (green) configurations over epoch of Experiment 3. **(C)** Intercepts (ms) for predictive (red) and random (green) configurations over epoch of Experiment 3.

For the analysis of mean RT data, trials with incorrect response and RTs below 200 ms or above 4000 ms (less than 0.6% of the trials) were again excluded. The mean RT values for predictive and random configurations with epochs for set sizes of 8 and 12 are shown in **Figure 4** (left and right panels, respectively). Three-way repeated ANOVA demonstrated significant main effects of configuration [*F*(1,20) = 9.304, *p* = 0.006, η^2^ = 0.318], indicating 29.59 ms faster RTs in predictive configurations than in random configurations; epoch [*F*(6,120) = 31.969, *p* < 0.001, η^2^ = 0.615], indicating 226.98 ms reduction of RTs over time; and set size [*F*(1,20) = 126.013, *p* < 0.001, η^2^ = 0.863], indicating 157.48 ms greater RTs for the larger set size.

The two-way interactions were significant for configuration × epoch [*F*(6,120) = 11.652, *p* < 0.001, η^2^ = 0.368]; and set size × epoch [*F*(6,120) = 5.030, *p* < 0.001, η^2^ = 0.201]; but not reliable for configuration × epoch. Moreover, the three-way interaction between configuration × epoch × set size was significant [*F*(6,120) = 3.423, *p* = 0.016, η^2^ = 0.146]. To further explore the contextual benefit in every epoch for set size 8 and 12, we used a *post hoc* simple effects analysis. For set size 8, the difference of configuration did not emerge at the first two epochs (*p*’s > 0.343). However, the contextual benefit of predictive configuration was obtained from the 3rd epoch to the last epoch [epoch 3: *F*(1,20) = 11.49, *p* = 0.003; epoch 4: *F*(1,20) = 5.51, *p* = 0.029; epoch 5: *F*(1,20) = 24.94, *p* < 0.001; epoch 6: *F*(1,20) = 6.53, *p* = 0.019; and epoch 7: *F*(1,20) = 8.09, *p* = 0.01], indicating that contextual benefit was obtained as the epoch progressed in the smaller set size. For set size 12, the RTs of predictive configuration was significantly greater than random configuration in the first epoch [*F*(1,20) = 9.75, *p* = 0.005]. The larger RTs for the predictive displays disappeared from the 2nd epoch to the 4th epoch (*p*’s > 0.310). In the later epochs the trend of larger RTs for the predictive display shown in the first epoch reversed into contextual benefit [epoch 5: *F*(1,20) = 18.76, *p* < 0.001; and epoch 7: *F*(1,20) = 23.87, *p* < 0.001], although no significant difference was obtained in the 6th epoch. Overall, contextual benefit became evident as we compared the earlier with later epochs for the larger set size. One way ANOVA for the Cueing effect of the learning epochs showed significant main effect of epoch [*F*(6,120) = 11.652, *p* < 0.001, η^2^ = 0.368], indicating no learning effect was obtained.

The slope as a function of configuration and epoch are shown in **Figure 4B**, the corresponding intercept are in **Figure 4C**. For the slope data, there was a significant main effect of epoch [*F*(6,120) = 5.030, *p* < 0.001, η^2^ = 0.201], the main effect of configuration and the two-way interaction were not reliable. For the slope data, there was a significant main effect of epoch [*F*(6,120) = 2.577, *p* = 0.040, η^2^ = 0.114], the main effect of configuration and the two-way interaction were not reliable.

### Discussion

The purpose of Experiment 3 was to evaluate the possibility that tiny 2D displacement of stimuli affected the contextual cueing effect in Experiment 2. In predictive configurations, each item presented in both the left eye and right eye was synchronously displaced by a small amount, while disparity for each item was kept constant over blocks. The result of Experiment 3 showed that the interaction for configuration and epoch were significant both in set size 8 and set size 12, suggesting that the contextual cueing effect took place. Thus, a small displacement of the distracter items would not interfere with the stability of the global visual context.

## General Discussion

In this study, we performed three experiments to test whether binocular disparity of the items in a scene can contribute to the contextual cueing effect. Experiment 1 demonstrated contextual cueing effect when the spatial layouts of the distracter items were defined by both binocular disparity and 2D displacement. Experiment 2 ruled out the possibility that participants relied solely on the 2D structure in the presence of stereo cues. In particular, Experiment 2 showed that when the stereoscopic depth of the distracter items varied across blocks (i.e., contexts were no longer invariant in depth across blocks) but the 2D spatial relationship between distracters was largely held constant across blocks, the contextual cueing effect greatly diminished. Experiment 3 ensured that the tiny 2d displacements leading to the variation in disparities in Experiment 2 would not prevent the occurrence of the contextual cueing effect. Overall, we demonstrated that information about the binocular disparity of the search items in the scene were used together with the 2D information in obtaining the contextual cueing effect.

Compared to 2D presentation, visual information in a 3D environment becomes more complicated, which could lead to additional attentional divergence. For implicit learning of spatial layout, no prior study has identified the contribution of stereoscopic depth of the distractor items in the simultaneous invariance of 2D spatial information. Tsuchiai et al. (2012) demonstrated that contextual cueing effect could be generated when binocular disparity between search items were held constant over blocks for repeated display. However, they did not examine whether the disparity information was actually used for implicit learning.

In a 3D environment, depth information plays a unique role in visual search (Nakayama and Silverman, 1986) and participants can intentionally use disparity information to restrict attention to a specific surface, in much the same way as they use information about color, motion, etc (Chun and Jiang, 1999; Huang, 2006; Akhavein and Farivar, 2017). Our visual system interprets specific loci of objects and their associations by stereoscopic depth, which cannot be represented in 2D space. Through 2D presentation, we can only use monocular cues, such as size, occlusion, or shading, etc., to illustrate 3D spatial relationships. In the current experiments, however, extraneous monocular cues were absent, or held constant across conditions. Participants could not judge depth relations by the monocular information. Thus, if participants performed the task by only adopting a 2D search strategy, the 3D spatial relations between items would be disregarded. The results of Experiment 2 in our study showed that when the stereoscopic depth information varied across blocks in the predictive display, little contextual cueing was obtained, suggesting invariant depth information (together with invariant 2D information) would contribute to the learning the contextual association.

In the early phase, there was a negative contextual cueing in the study. Greater benefit in random configurations than in predictive configurations was found both in the first two epochs for Experiment 1. The same thing was happened in the first epoch and 3rd epoch for Experiment 3, resulting in a significant three-way interaction in Experiment 3. This might be caused by the variation of configuration. To obtain the contextual cueing effect with the smallest variance between participants, the predictive configurations were generated in advance, and we ruled out configurations in which the target was easily searched. All the participants had the same predictive displays.

It is noted that the contextual cueing, differences between predictive and random displays, was less in our experiments than previous studies. For example, the contextual cueing in Experiment 3 was about 50 ms, by comparison with almost 100 ms in the study of Chun and Jiang (1998). Moreover, the contextual cueing in set size 12 was less than in set size 8 in our three experiments. This was different from previous studies (Chun and Jiang, 1998). This might the major cause for the inconsistent findings on the RT × Set Size data from previous studies (Chun and Jiang, 1998; Kunar et al., 2007). The perception of depth might be a cause that suppressed contextual cueing effect when visual context learned by non-semantic memory. In our experiments, the contexts were consisted by an array of letters, by which the regularities between target and distracters were arbitrary and non-semantic. When contexts viewed by stereovision, these regularities multiplied and non-semantic, thus weaken the power of contextual cueing effect.

Since the initial work of Chun and Jiang (1998), most of the previous studies use a 2D display of letters. The association between the location of the target and the location of the distractor items formed the base of associative learning. In addition to 2D spatial location, what other information could contribute to the learning of the target-context association is an interesting question. In the layout of distractor letter L, the contextual cueing effect could be obtained even when the orientations of the distractors in repeated configurations were randomized across blocks (Johnson et al., 2007; Pollmann and Manginelli, 2009; Schankin and Schubo, 2009, 2010; Tseng and Lleras, 2013). This result suggested that with the invariant location information, the identity information of the distracters (defined by orientation) could be ignored and did not prevent the learning of the target-distractor location association. It is important to point out that that in this case, some distractors shared the same orientation, thus the identity information might not be unique enough to be informative.

While participants can ignore the variant identity information of the individual distractors when picking up the invariant spatial relation between target and distractor, the current study showed that the stereoscopic depth information of the individual distractors cannot be ignored (Experiment 2). Participants might have automatically incorporated the stereoscopic depth information with the 2D location information when viewing the layout. This result is reminiscent of the findings of He and Nakayama (1992) who demonstrated that during visual search, participants prioritized stereoscopic depth information over 2D feature information. Based on the results of previous research dealing with the binding of identity with location and our current study, it is likely that in the contextual cueing paradigm, when 2D layout information is invariant, participants can ignore competing variant identity information, but they prioritize stereoscopic depth information in learning the layout.

In sum, the present study reexamined contextual cueing effect when the visual context was defined by binocular disparity. The contextual cueing effect occurred when the 3D structure was predictive and disappeared when the information of stereoscopic depth was variant despite predictive 2D layouts. Stereoscopic depth information should be considered as an integrated part of the spatial information that define a 3D layout. The current study demonstrates that in a 3D space, the contextual cueing effect could rely on both 2D and depth information in the context.

## Author Contributions

GZ and QL designed and implemented the experiments. JM, QZ, and ST, collected the data. GZ analyzed the data and created the initial draft of the manuscript. QL and H-jS revised the paper. All authors reviewed the manuscript.

## Conflict of Interest Statement

The authors declare that the research was conducted in the absence of any commercial or financial relationships that could be construed as a potential conflict of interest.
